# Induction of Male Courtship Behavior by Olfactory Cues Released From Ovulating Female in the Medaka, *Oryzias latipes*

**DOI:** 10.1002/jez.70017

**Published:** 2025-07-30

**Authors:** Yasunori Koya, Megumi Kodama, Tsukasa Maruyama, Yuki Kondo

**Affiliations:** ^1^ Faculty of Education Gifu University Gifu Japan; ^2^ Laboratory of Animal Sociology, Department of Biology, Graduate School of Science Osaka Metropolitan University Osaka Japan

**Keywords:** female receptivity, ovulation, releaser pheromone, urine

## Abstract

Chemical communication between males and females is important for successful reproduction of animals. The activation of male courtship behavior by female olfactory cues has only been demonstrated in a limited number of teleost species. In the present study, we showed that in the model animal medaka, courtship behavior in males are activated by olfactory cues released by females and that at least one of them is released through urine. Male medaka perform a series of courtship behaviors, such as “following” (chasing the female), “positioning” (positioning slightly behind the female), and “quick‐circle” (performing a somersault around the female's nose), before spawning. Males rarely showed courtship behavior toward post‐spawning females; however, in the waters that had been reared ovulating female or other mating pairs, males significantly increased their following toward post‐spawning females. Furthermore, males tended to make more frequent quick‐circle toward post‐spawning females in the water of other mating pairs had been spawned in it. When males were paired with post‐spawning females in water supplemented with ovulating‐female urine, only the frequency of quick‐circle increased significantly. This suggested that the urine of ovulating females contains a component that induces the quick‐circle and that the component inducing the following is a different component released via a route separate from urine. Ovulating females store more than ten times the amount of urine in their bladders than non‐ovulating females and males, and releasing this urine during male courtship behavior may increase male motivation.

## Summary

- Ovulating female medaka release olfactory cues that induce courtship behavior in males.
- One component of olfactory cues is contained in the urine of females, stimulating “quick‐circle” of males.
- Ovulating females store large amounts of urine in their bladders.

## 1 Introduction

In sexually active animals, mutual receptivity between males and females and mate choice are important for successful sexual reproduction (Anholt et al. 2020). To reproduce successfully, male of oviparous animals must determine whether a female is in a state where she can lay eggs, that is, whether she is ovulating, using a variety of information (Maruska and Butler 2021).

In some teleost species, males use the chemicals released by females to determine whether they are sexually receptive or ready to spawn. F‐type prostaglandins released from ovulating females induce spawning behavior in male goldfish, *Carassius auratus*, and zebrafish, *Danio rerio* (Sorensen et al. 1988; Yabuki et al. 2016). It has been suggested that male tilapia, *Oreochromis mossambicus*, discern the reproductive status of females using chemicals released into the water via their urine and feces (Miranda et al. 2005; Ashouri et al. 2023). Female masu salmon (*Oncorhynchus masou*) communicate their ovulation to males through the odor of kynurenine, a tryptophan metabolite present in their urine (Yambe et al. 2006).

Medaka (*Oryzias latipes*) is a teleost fish that is widely used as a model organism, and the following knowledge has been obtained about its reproduction. Medaka lay eggs almost every day in an environment suitable for breeding (high water temperature and long day length) (Shibata et al. 2011). During the breeding season, males always have sperm, and when they meet a female capable of spawning, they will encapsulate the female after a series of courtship behaviors and release sperm at the same time as the female releases her eggs. In captivity, one male can spawn with multiple females in a day (Kondo et al. 2025a). If the female is in good nutritional condition, maturation and ovulation of oocytes will occur late at night every day (Shibata et al. 2011), and if ovulating females meet a male, they spawn only once a day (Grant and Green 1996). The female cannot release the eggs without the body vibrations from the male (Egami and Nambu 1961). They produce anywhere from a dozen to several dozen eggs in one spawning session, and generally all eggs that are ovulated are released, leaving none behind (Kondo et al. 2025a). After spawning, they dangle the fertilized eggs from their reproductive opening for a while, and then within a few hours, they become entangled in aquatic plants or other objects (Kobayashi et al. 2012).

Several observations and experiments have been conducted in medaka to resolve how males determine whether females are in a state that they can lay eggs (ovulating). Ono and Uematsu (1968a) demonstrated that males actively engaged in courtship behavior toward models whose morphology resembled that of females but did not engage in courtship behavior toward models that did not, demonstrating the importance of vision in medaka reproduction. In contrast, Egami and Nambu (1961) showed that spawning occurs even when the eyes of male medaka were removed, and Kondo et al. (2025b) clarified through field observations that wild medaka spawn late at night. These reports indicate that visual information is not a prerequisite for medaka reproduction. Hayakawa et al. (2012) showed that when the nostrils of male medaka were sealed with adhesive, spawning was no longer observed even in pairs that had spawned daily before the treatment, suggesting that male olfactory information is essential for spawning in medaka. However, it remains unclear at what stage of egg‐laying the olfactory information is required.

During daily spawning, male medaka exhibit courtship behavior by sensing some kind of information about whether the female is ready to spawn, that is, whether she is ovulating. Based on the results of some of the studies mentioned above, it appears that visual information is not essential in the case of medaka for males to determine whether or not a female is in a state where she can lay eggs. Rather, olfactory information, that is, the chemical cues released by the female, are important. The reason why male medaka, whose sense of smell was blocked, did not spawn in the study by Hayakawa et al. (2012) can be interpreted as the males being unable to perceive the sex pheromones released by the females. However, no study has demonstrated the existence of olfactory cues in medaka that signals females' readiness to lay eggs to males.

In the present study, we aimed to clarify that olfactory cues emitted by females are necessary for male medaka fish to court females. Therefore, we first investigated whether males recognize that females are ovulating and court females accordingly. As a result, we confirmed that males court females during ovulation, but not after females have already laid eggs. We next investigated whether males court females after spawning, using various stimulus water (containing odor of ovulating females or other females before and after spawning, and containing urine from ovulating females) on the assumption that ovulating females emit olfactory cues that induce males' courtship behavior. To demonstrate the importance of urine as an olfactory cue emitted by females, we investigated whether ovulating females store more urine in their bladders than usual.

## 2 Methods

### 2.1 Breeding of Experimental Fish for Behavioral Experiments

Wild‐type medaka, *Oryzias latipes*, were collected from small waterways around Gifu University, Gifu, Japan, and were reared for behavioral experiments in three holding tanks (each 180 cm in length, 90 cm in width, and 80 cm in height; approximately 1200 L) set up outdoors at Gifu University. The water of holding tanks was not temperature regulated (average ambient temperature varied between approximately 20°C–31°C). The fish were kept without filtration or aeration, and were fed granular feed for fish larvae (“Otohime B2” MARUBENI NISSHIN Co. Ltd., Tokyo, Japan) once or twice daily during the breeding season (May–August). If kept under these conditions, most adult females will spawn every day during the breeding season. Adult female and male [28.3–41.9 (mean 34.4 mm) and 27.7–37.9 (mean 32.0) mm in total length, respectively] were selected for the experiment. The experiments were conducted over two consecutive days. On the first afternoon, males and females were randomly scooped from the holding tanks and kept individually in cubic isolation tanks with a side length of 15 cm (approximately 3.2 L capacity) set up indoors. The water temperature was not regulated (the room temperature was adjusted to 25°C using an air conditioner) and the fish were kept without filtration or aeration. On the morning of the second day, males and females were introduced into an observation tank of the same size and rearing conditions as the isolation tank set up indoors, and their behavior was observed. When pairing a male and female, we always used individuals from different holding tanks to ensure that the males and females met for the first time. Individuals used for observation were transferred to separate holding tanks and were not reused. The water used in the isolation and observation tanks was tap water stored for more than 1 day.

All animal experiments were approved by the Institutional Animal Experiment Committees of Gifu University and Osaka Metropolitan University.

### 2.2 Recording and Counting of Courtship Behavior

**Figure 1:**
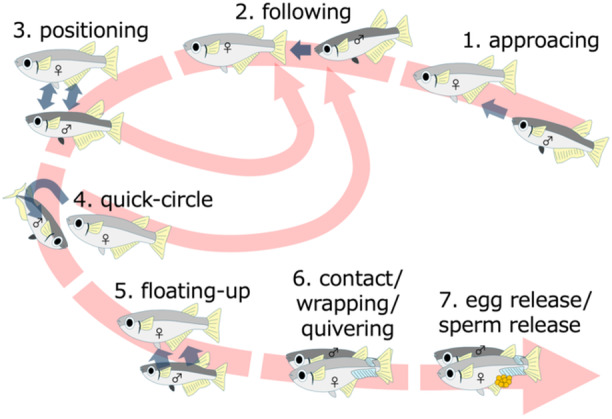
Schematic diagram of the sequence of courtship behavior in medaka fish by Ono and Uematsu (1957) and Hayakawa et al. (2012).

The courtship behavior of medaka (Figure 1) is defined as follows by Ono and Uematsu (1957) and Kobayashi et al. (2012): When a male finds a female, male approaches female (“approaching”), and after chasing her for a while (“following”), male stops slightly below her (“positioning”). After repeating these actions, the male swims in circles around the tip of female's snout (“quick‐circle”). When the female raises her head, the male slowly rises from the side (“floating‐up”), holds her with his anal and dorsal fins (“contact/wrapping“). If the female does not move, the male shakes his body while vigorously moving his pectoral and caudal fins (“quivering”), releasing the eggs and sperm, and completing spawning. In the present study, we counted the “following,” “positioning,” and “quick‐circle” events and the time it took for male to the first following, which are relatively easy to distinguish from video images.

Immediately after placing a pair of males and females in the observation tank, the courtship behavior of the medaka was filmed from the front with a video camera (HDR‐CX700, Sony Corporation, Tokyo, Japan) for approximately 60 min. For the following and positioning behaviors, the number of events after the start of pairing was recorded for 10 min. Because quick‐circle occur later in courtship behavior, the number of times they occur was recorded for up to 40 min after pairing. When a paired ovulating female and male spawned before the specified observation time, the number of following and positioning behaviors was converted to the number of times per 10 min [number of following or positioning behaviors/time taken for spawning (seconds) × 600 (seconds)]. The quick‐circle behavior was similarly converted to the number of times during 40 min.

### 2.3 Observation of Courtship Behavior Toward Ovulating Females and Post‐Spawning Females

We observed the behavior of males toward ovulating females that were able to spawn (*N* = 5) and post‐spawning females that were unable to spawn (*N* = 6) to investigate whether males sense that females are able to spawn and engage in courtship behavior toward them. Females and males from different holding tanks were transferred to an isolation tank on the first day, and the females that had spawned in the isolation tank on the second day were used as post‐spawning females. Before pairing the post‐spawning females with new testing males, the eggs laid by the females were removed, the females' bodies were thoroughly rinsed with distilled water and left for 1 h. This is to eliminate the influence of other excretions, such as skin mucus, urogenital fluids, or feces, from the post‐spawning females on the behavior of the males. Post‐spawning females and males were placed in an observation tank containing tap water, and their behavior was videotaped.

### 2.4 Observation of Courtship Behavior in “Stimulus Water”

We investigated whether males engaged in courtship behavior toward the post‐spawning females in “stimulus water” that may contain some olfactory cues to verify the hypothesis that “males are determining whether or not females are able to spawn by detecting chemical signals from females.” The post‐spawning females used in the following behavioral observations were individuals that had been paired with another male just before behavioral observation and allowed to spawn, after which the eggs were removed, the female's body was thoroughly rinsed with distilled water, and left for 1 h. This is to eliminate the influence of other excretions, such as skin mucus, urogenital fluids, or feces, from the post‐spawning females on the behavior of the males.

#### 2.4.1 “Ovulating‐Female Water” and “Male Water”

**Figure 2:**
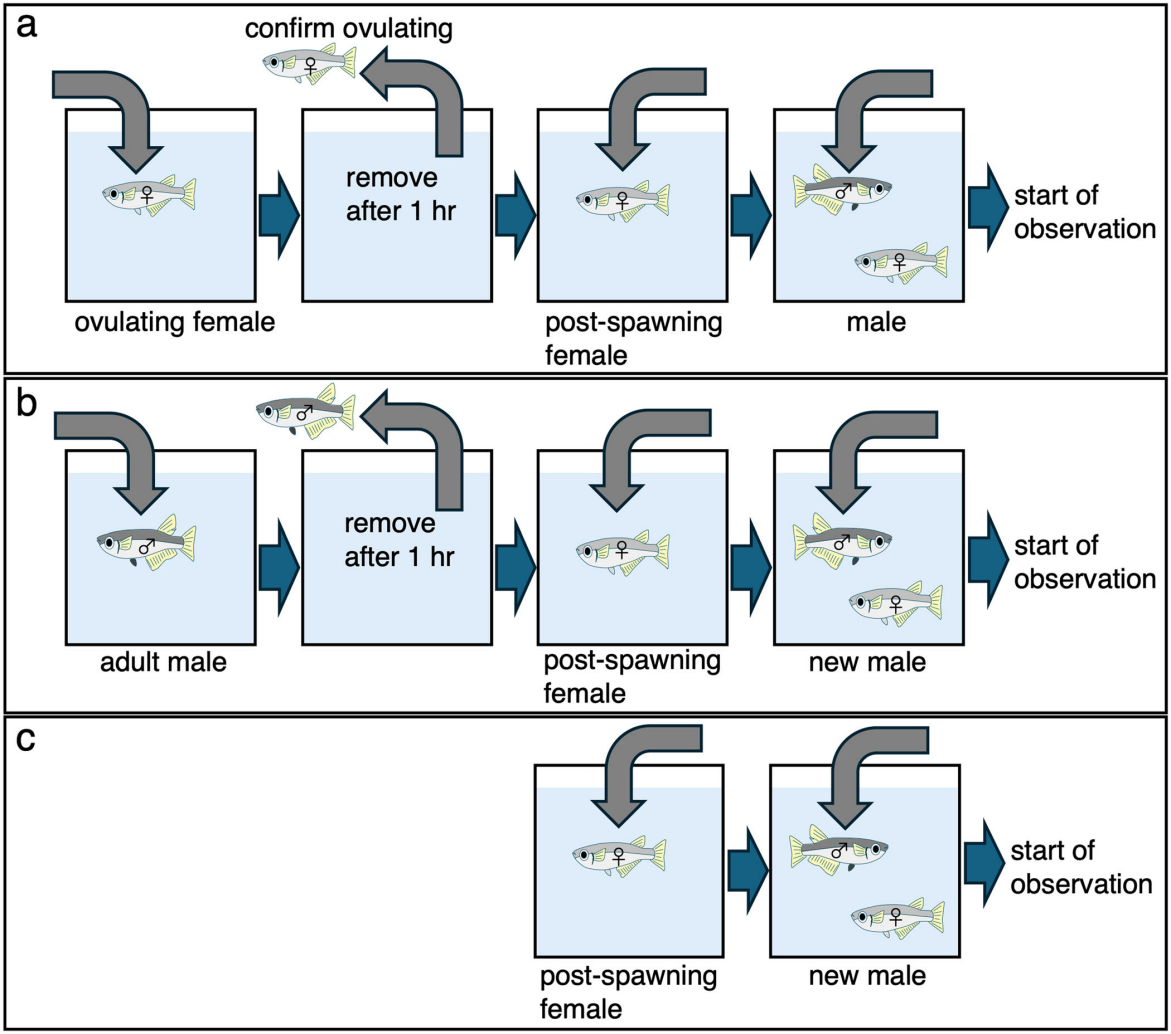
Flow chart for conducting stimulus water experiments using “ovulating‐female water” (a), “male water” (b), and “tap water” (c).

The rearing water of ovulating female was used as the stimulus water (“ovulating‐female water”; Figure 2a; *N* = 7). To obtain the “ovulating‐female water”, an ovulating female was introduced into the isolation tank and kept for 1 h. After that, the ovulating female was removed from the tank and a new testing male was paired with a post‐spawning female in the tank to observe the male's courtship behavior (Figure 2a). To confirm whether the females used as “ovulating females” in this experiment had truly ovulated, this female was removed from the isolation tank and then placed in another isolation tank with another male, and it was confirmed that she had subsequently laid eggs (she was ovulating). The rearing water of male (“male water”; Figure 2b; *N* = 7) and untreated water (“tap water”; Figure 2c; *N* = 6) were used as controls. To obtain the “male water”, a male was introduced into the isolation tank and kept there for 1 h. After removal, a new male was paired with a post‐spawning female in the same tank and the male's courtship behavior was observed (Figure 2b).

#### 2.4.2 “Pre‐Quick‐Circle Water” and “Post‐Spawning Water”

**Figure 3:**
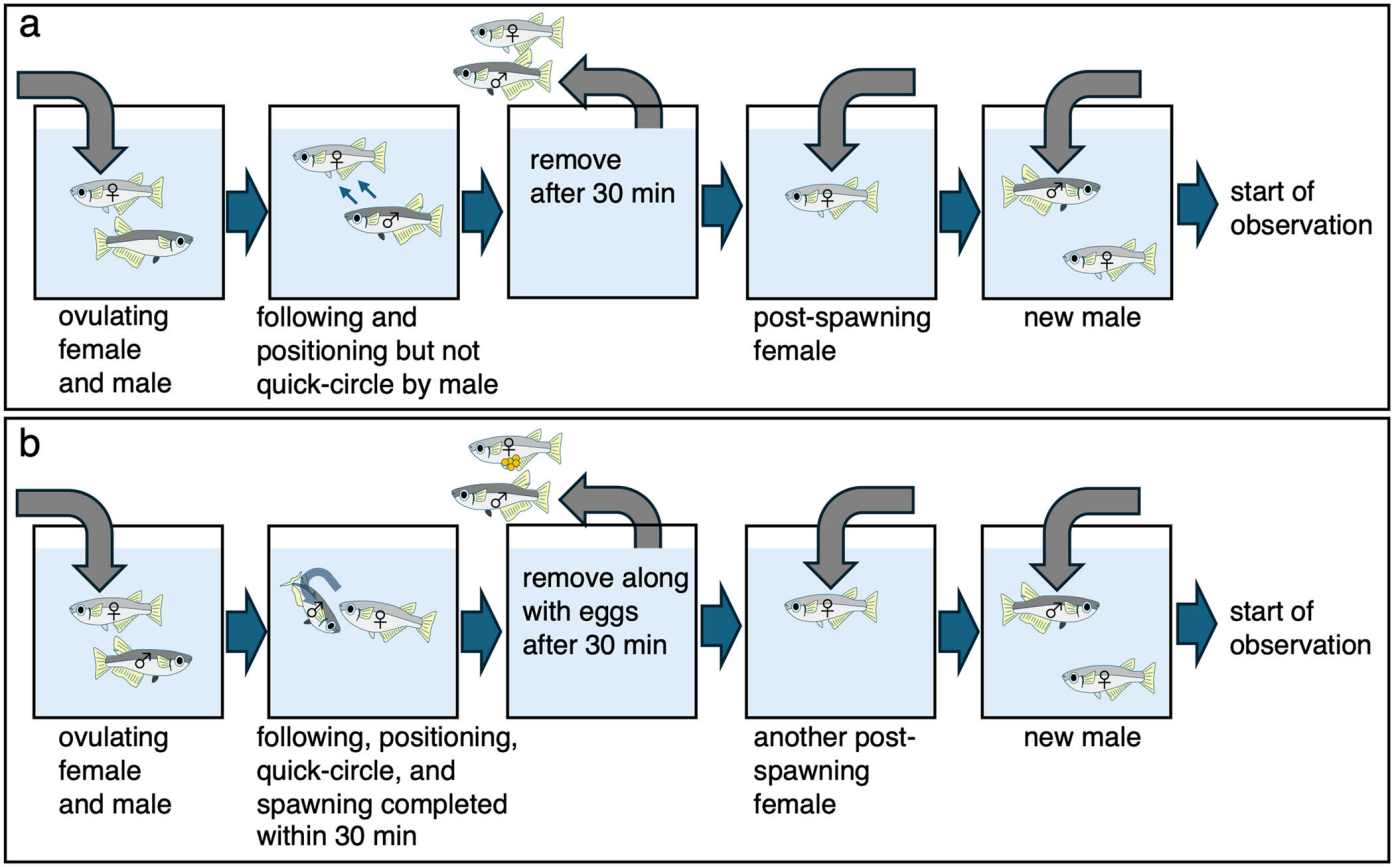
Flow chart for conducting stimulus water experiments using “pre‐quick‐circle water” (a) and “post‐spawning water” (b).

In medaka, male courtship behavior has several stages (following, positioning, quick‐circle etc.), which change over time depending on the interaction with the female (Ono and Uematsu 1957; Figure 1). This suggests that the effect of the olfactory stimuli emitted by the female is enhanced or the components and functions of the olfactory stimuli change depending on the interaction with the male. Therefore, to examine whether the effects and properties of olfactory stimuli released by females changed as male courtship behavior progressed, we used as stimulus water in which the males had not performed the quick‐circle (“pre‐quick‐circle water”; Figure 3a; *N* = 5) and the water in which the males had performed the quick‐circle, followed by wrapping, quivering, and spawning (“post‐spawning water”; *N* = 6). To obtain the “pre‐quick‐circle water”, we paired an ovulating female with a non‐test male in an isolation tank, confirming that they were engaging in normal courtship behavior such as following and positioning, and then, we used the water from those specimens when no quick‐circle was observed after 30 min for the experiment (Figure 3a). After removing the males and females from the tank after 30 min, we paired a post‐spawning female with a new testing male in the same tank and observed their behavior.

To obtain the “post‐spawning water”, we placed an ovulating female and a non‐test male in an isolation tank, confirmed that they were performing normal courtship behavior, and used the rearing water that led to spawning within 30 min (Figure 3b). After removing the males and females from the tank, we paired a new post‐spawning female with a new testing male in the same tank and observed their behavior. As an control, the behavior of males toward post‐spawning females in untreated water (“tap water”; same as Figure 2c; *N* = 6) was used.

### 2.5 Urine‐Added Water

The behavior of males toward post‐spawning females was observed when urine from ovulating females was added. The experiment was conducted over 2 consecutive days. On the first afternoon, females were randomly scooped from the holding tanks and transferred to each isolation tank. On the morning of the second day, ovulating females were anesthetized with 200 mg/l ethyl 3‐aminobenzoate methanesulfonic acid salt (MS‐222; Sigma‐Aldrich, St. Louis, MO, USA) and their abdomens were cut open to expose the bladder. A small triangular piece (approximately 1 cm on each side) of filter paper (No. 2, Advantec Toyo Kaisha Ltd., Tokyo, Japan) was pressed against the bladder, dissected bladder with scissors, and the urine that emerged was absorbed by the filter paper. The filter paper was placed in an observation tank filled with tap water, and the resulting solution was used as “urine‐added water.” Post‐spawning females were paired with males in an observation tank containing urine‐added water, and their behavior was observed thereafter. As control (“tap water”), the behavior of males toward post‐spawn females was observed in an observation tank filled with tap water containing only small triangular piece of unused filter paper.

### 2.6 Measurement of Urine Volume

Commercially available orange‐type medaka were kept in eight tanks (each 90.5 cm length, 60.5 cm width, 21 cm height; about 104 L) set up indoors with approximately 100 individuals in each tank. Even in the nonbreeding season, medaka will begin spawning within a few weeks if the water temperature and photoperiod are properly adjusted. A filtration device was installed in the tank, and the fish were kept at 26 ± 1°C for 1 month under a 14L10D photoperiod (light period: 08:00–22:00, dark period: 22:00–08:00), and then used for a urine volume measurement experiment. The fish were fed Tetramine (Tetra, Melle, Germany) three times daily. The tanks were observed every morning, and urine volume was measured after confirming that approximately half of the females in the tank had laid eggs hanging from their abdomens. Fifteen females and five males were isolated in separate tanks the day before the measurement to prevent spawning, and measurements were carried out the next morning. After anesthetizing the fish, the moisture on the body surface was wiped off, and the body weight was measured. The abdomen was cut open using scissors to expose the bladder. A microcapillary tube (0.5 × 0.2 × 120 mm; 0.0462 g on average weight) (Fujirika Kogyo Co. Ltd., Osaka, Japan) whose weight had been measured in advance was attached to the bladder, the bladder was dissected with scissors, and urine was absorbed into the capillary tube. Dissection of the experimental female fish revealed that eight had ovulated and five had not. The weight of the urine was calculated by measuring the weight of the capillary tube that had absorbed the urine. Urine weight was divided by body weight and multiplied by 100 to obtain the urine‐somatic index. Measurements were performed on five mature males, five non‐ovulating females, and eight ovulating females. The measurement limit of the balance (electromagnetic force balancing system) (XFR‐135; Shinko Denshi Co. Ltd., Tokyo, Japan) was 0.00001 g. When the urine weight was below the measurement limit, it was treated as 0.00001 g for statistical analysis.

### 2.7 Statistical Analysis

All numerical data were expressed as mean ± standard error of the mean (SEM). A box plot was created from the obtained data for behavioral experiments, and values that did not fall between (1st quartile–1.5 × interquartile range) and (3rd quartile + 1.5 × interquartile range) were considered outliers.

All statistical analyses were conducted using R version 4.4.1 (R Core Team 2024). Non‐parametric tests were employed throughout the study. The Mann‐Whitney U test was used for experiments involving two‐group comparisons. This test was applied to the observation of courtship behavior toward ovulating females and post‐spawning females (Section 2.3), as well as the urine‐added water experiment (Section 2.5). The Kruskal‐Wallis test was performed for experiments involving three‐group comparisons. This test was used to analyze the “ovulating‐female water” and “male water” experiments (Section 2.4.1), the “pre‐quick‐circle water” and “post‐spawning water” experiments (Section 2.4.2), and the measurement of urine volume comparing ovulating females, non‐ovulating females, and males (Section 2.6). When the Kruskal‐Wallis test revealed significant differences among groups, the Steel‐Dwass test was conducted as a post‐hoc multiple comparison test to identify which specific groups differed from each other. A significance level of *p* < 0.1 indicated a tendency, and *p* < 0.05 indicated a significant difference.

## 3 Results

### 3.1 Male Courtship Behavior Toward Ovulating and Post‐Spawning Females

Observations of male behavior toward ovulating females in the five pairs revealed that the times required for spawning were 840, 1503, 9711, 1012, and 497 s, respectively, and the time required for spawning from pairing varied among pairs.

**Figure 4:**
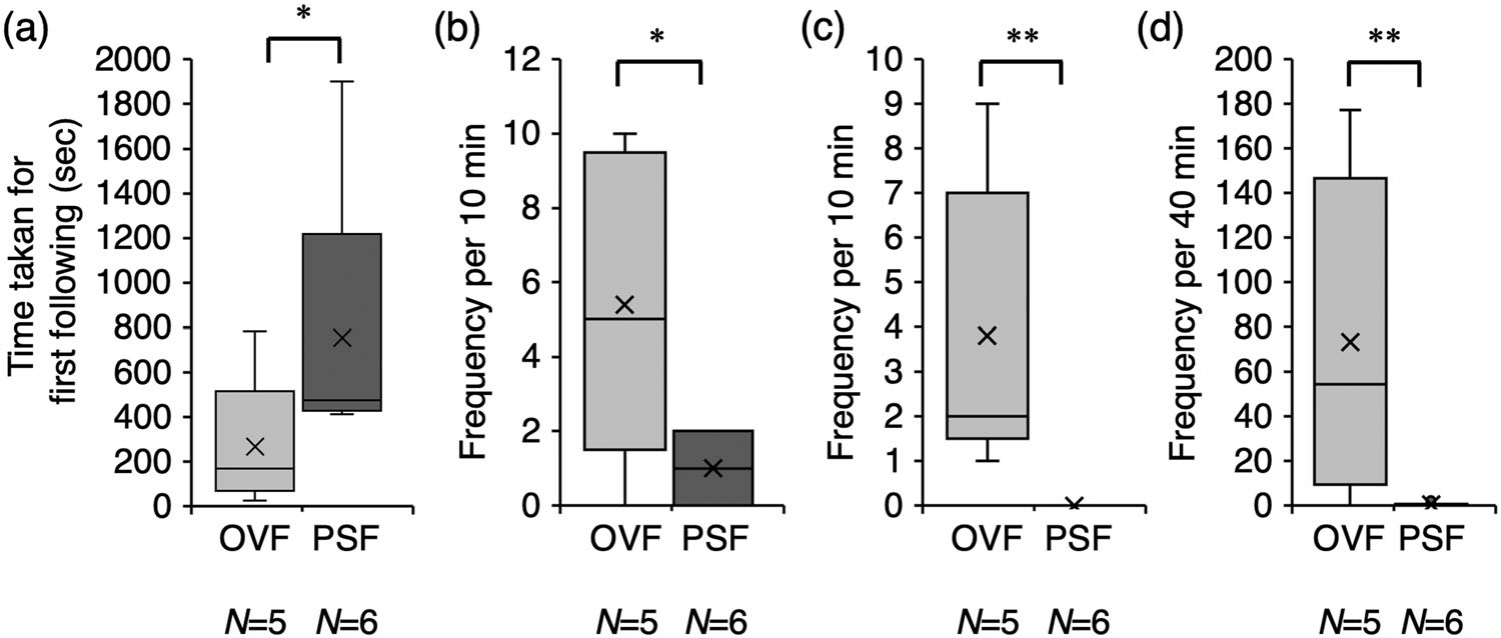
Box plots of the time taken for first following behavior (a), and frequency of following (b), positioning (c), and quick‐circle (d) of males toward ovulating females (OF) and post‐spawning females (PSF) in courtship behavior of medaka, *Oryzias latipes*. × marks indicate the average value. *p < 0.1, **p < 0.05 (Mann‐Whitney U test).

The time taken for the first following behavior by males toward ovulating females ranged from 25 to 783 s (*N* = 5), with four males performing their first following within 5 min. In contrast, the time taken for the first following of females ranged from 414 to 1900 s (*N* = 6), with four males taking longer than 6 min. The mean time taken for the first following behavior by males (Figure 4a) toward ovulating females was 268 ± 133.8 s, which tended to be shorter than the time taken toward post‐spawning females (754 ± 287.1 s) (Mann‐Whitney U test; *p* = 0.095).

The mean frequencies of followings by males (Figure 4b) toward ovulating females and post‐spawning females were 5.4 ± 1.86 and 1.0 ± 0.4, respectively, with the former tending to be more frequent than the latter (Mann‐Whitney U test; *p* = 0.079).

The mean frequency of positionings by males (Figure 4c) toward ovulating females was 3.8 ± 1.5, whereas none (0 times) was observed toward post‐spawning females; a significant difference between the two (Mann‐Whitney U test; *p* = 0.004).

The mean frequencies of quick‐circle by males (Figure 4d) toward ovulating females and post‐spawning females were 73.3 ± 32.6 and 0.3 ± 0.3, respectively; a significant difference between the two (Mann‐Whitney U test; *p* = 0.029). In the observations of ovulating females and males, males continued to engage in courtship behaviors, such as quick‐circle toward post‐spawning females, even after spawning in all pairs.

### 3.2 Male Courtship Behavior Toward Post‐Spawning Females in Ovulating‐Female Water and Male Water

**Figure 5:**
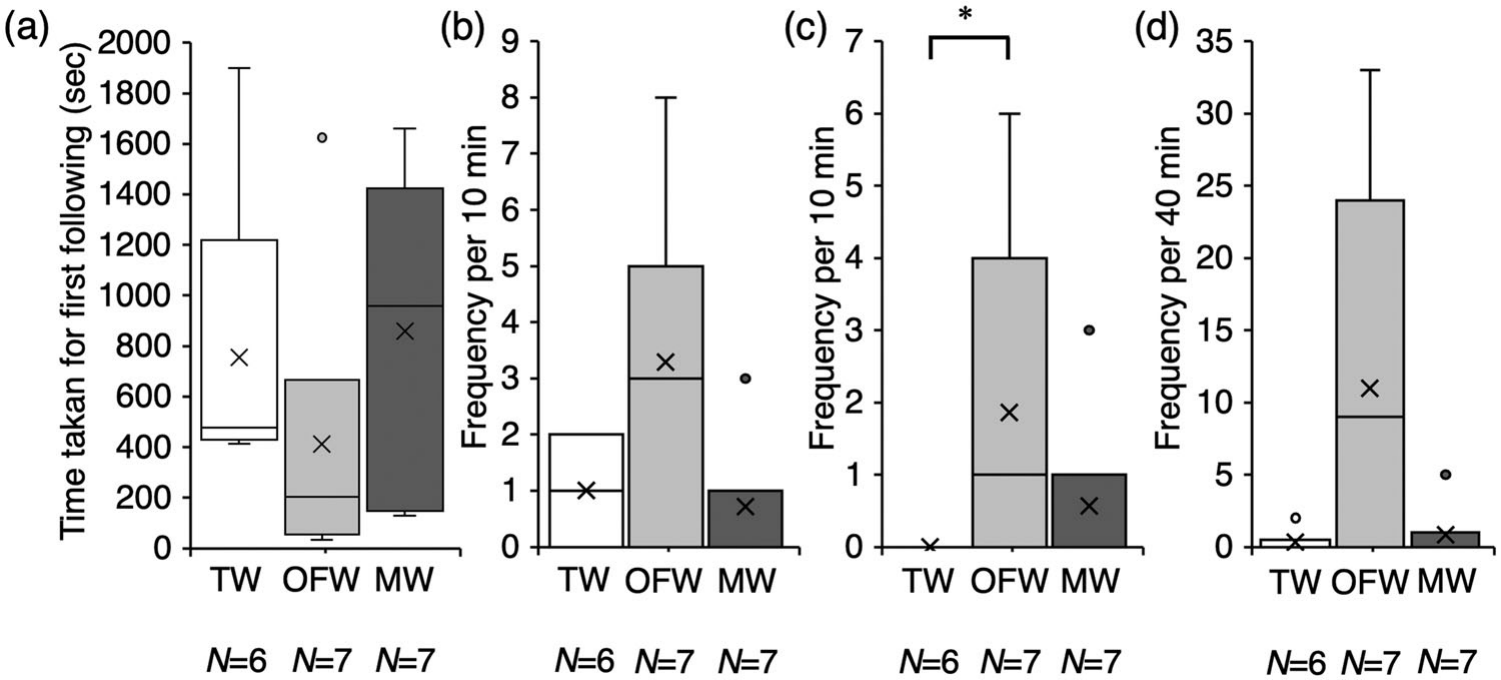
Box plots of the time taken for first following behavior (a), and frequency of following (b), positioning (c), and quick‐circle (d) of males toward post‐spawning females in “tap water” (TW), “ovulating‐female water” (OFW), and “male water” (MW) in courtship behavior of medaka, *Oryzias latipes*. × marks indicate the average value. *p < 0.1, **p < 0.05 (Steel–Dwass test).

The time taken for the first following behavior by males toward post‐spawning females in ovulating‐female water ranged from 33 to 1624 s (*N* = 6), with five males performing their first following within 6 min. In contrast, the time taken for first following toward post‐spawning females in male water ranged from 128 to 1661 s (*N* = 7), with five males taking longer than 9 min to follow first. The mean times taken for the first following behavior by males (Figure 5a) toward the post‐spawning female in tap water, ovulating‐female water, and male water were 754 ± 287.1, 412 ± 247.1, and 859 ± 249.8 s, respectively, and no significant differences were observed between them.

The mean frequencies of following by males toward post‐spawning females (Figure 5b) in tap water, ovulating‐female water and male water were 1.0 ± 0.4, 3.3 ± 1.0, and 0.7 ± 0.4, respectively, and no significant differences were observed between them.

The mean frequencies of positioning by males toward post‐spawning females (Figure 5c) in tap water, ovulating‐female water and male water were 0, 1.9 ± 0.9 and 0.5 ± 0.4, respectively. The Kruskal–Wallis test revealed a tendency for differences to be observed (*p* = 0.082), with the frequency in ovulating‐female water tending to be greater than that in tap water (Steel‐Dwass test; *p* = 0.079).

The mean frequencies of quick‐circle by males toward post‐spawning females (Figure 5d) in tap water, ovulating‐female water, and male water were 0.3 ± 0.3, 11.0 ± 4.9, and 0.9 ± 0.7, respectively, and no significant difference was observed between them.

### 3.3 Male Courtship Behavior Toward Post‐Spawning Females in Pre‐Quick‐Circle Water and Post‐Spawning Water

**Figure 6:**
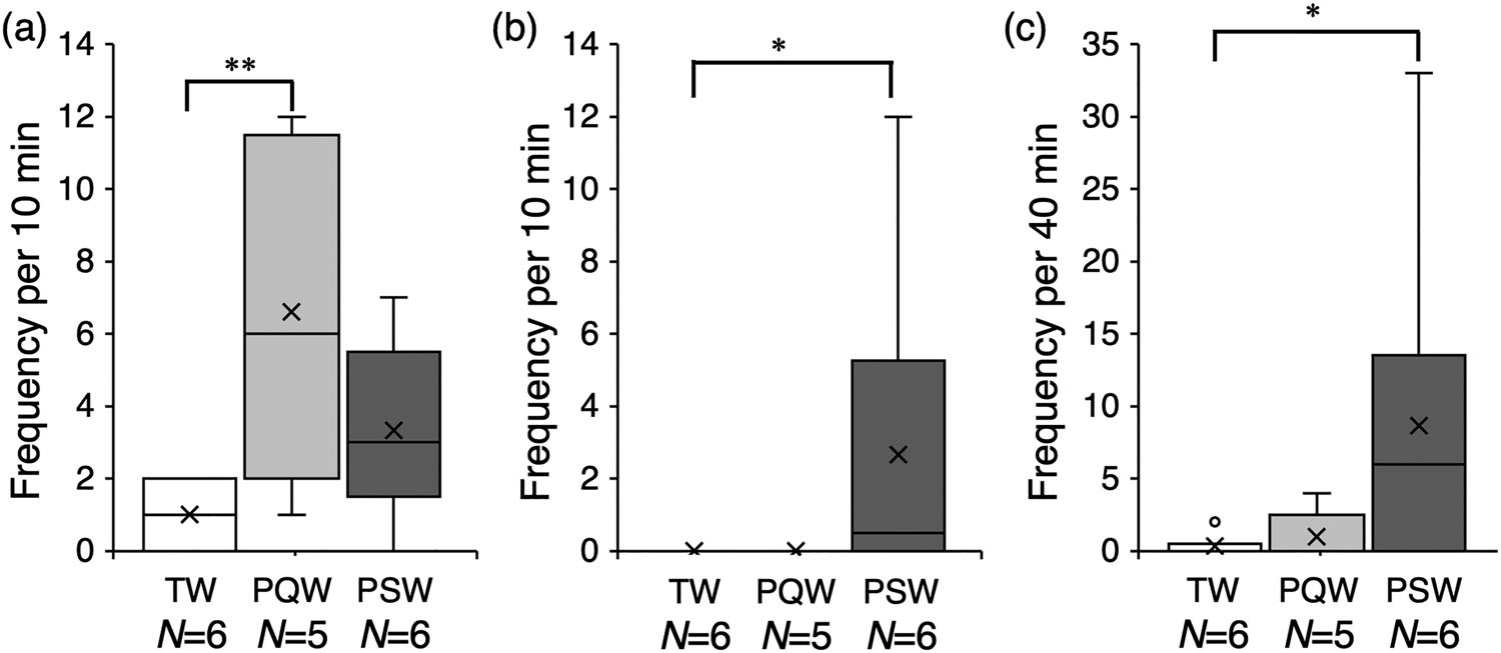
Box plots of the frequency of following (a), positioning (b), and quick‐circle (c) of males toward post‐spawning females in “tap water” (TW),”pre‐quick‐circle water” (PQW), and “post‐spawning water” (PSW), in courtship behavior of medaka, *Oryzias latipes*. × marks indicate the average value. *p < 0.1, **p < 0.05 (Steel–Dwass test).

The mean frequencies of following by males toward post‐spawning females in tap water, quick‐circle water, and post‐spawning water (Figure 6a) were 1.0 ± 0.4, 6.6 ± 1.9, and 3.3 ± 1.0, respectively. The Kruskal–Wallis test revealed significant differences (*p* = 0.036), with the frequency in pre‐quick‐circle water being significantly greater than that in tap water (Steel‐Dwass test; *p* = 0.036).

The mean frequencies of positioning by males toward post‐spawning females in tap water, pre‐quick‐circle water, and post‐spawning water (Figure 6b) were 0, 0, and 2.7 ± 1.9, respectively. The Kruskal–Wallis test revealed a tendency for differences (*p* = 0.045), with the frequency in post‐spawning water tending to be greater than that in tap water (Steel‐Dwass test; *p* = 0.085).

The frequencies of quick‐circle by males toward post‐spawning females in tap water, pre‐quick‐circle water, and post‐spawning water (Figure 6c) were 0.3 ± 0.3, 1.0 ± 0.8, and 8.7 ± 5.0, respectively. The Kruskal–Wallis test revealed a tendency for differences (*p* = 0.087), with the frequency in post‐spawning water tending to be greater than that in tap water (Steel‐Dwass test; *p* = 0.096).

### 3.4 Male Courtship Behavior Toward Post‐Spawning Females With Urine From Ovulating Females

**Figure 7:**
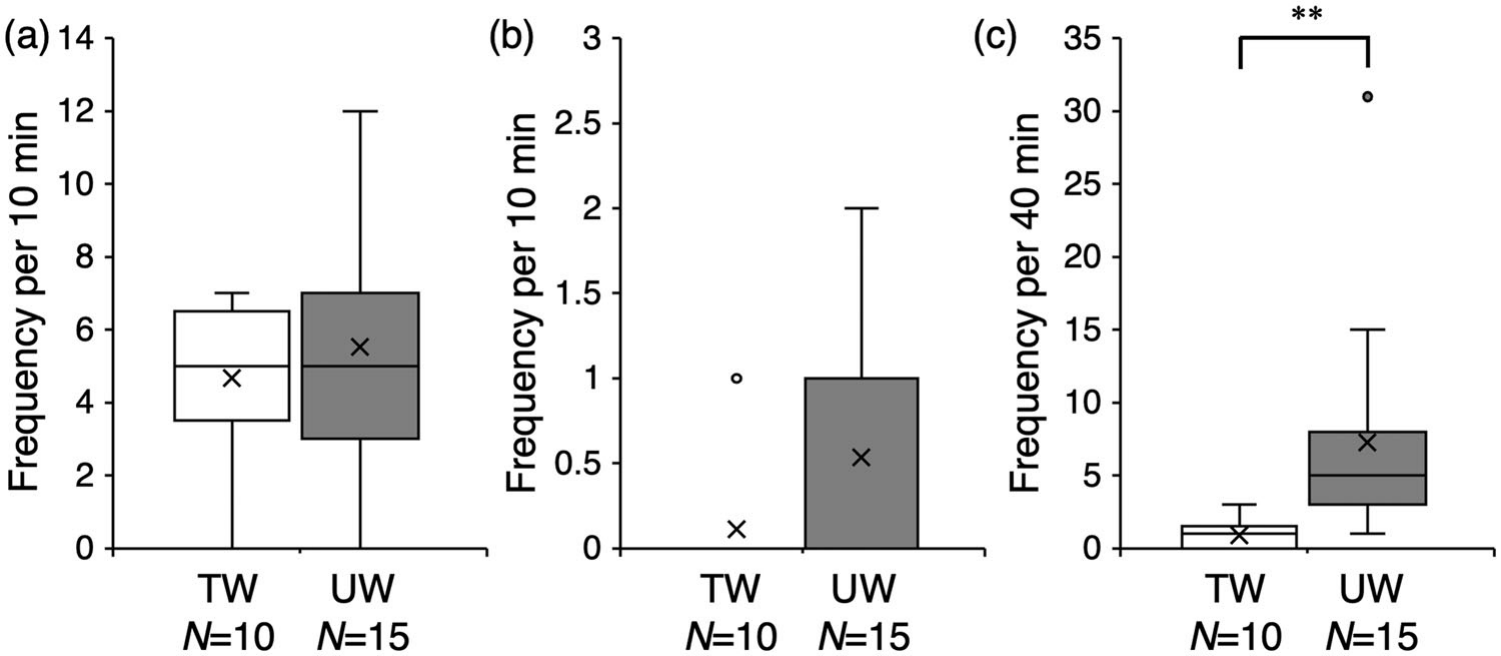
Box plots of the frequency of following (a), positioning (b), and quick‐circle (c) of males toward post‐spawning females in “tap water” (TW) and “urine‐add water” (UW), in courtship behavior of medaka fish, *Oryzias latipes*. × marks indicate the average value. **p < 0.05 (Mann‐Whitney U test).

The mean frequencies of following by males toward post‐spawning females (Figure 7a) in tap water and urine‐added water were 4.67 ± 0.73 and 5.53 ± 0.86, respectively, and no significant difference was observed between the two treatments.

The mean frequencies of positioning by males toward post‐spawning females in tap water and urine‐added water (Figure 7b) were 0.11 ± 0.11 and 0.53 ± 0.19, respectively, and no significant difference was observed between the two treatments.

The mean frequencies of quick‐circle by males toward post‐spawning females in tap water and urine‐added water (Figure 7c) were 0.89 ± 0.35 and 7.13 ± 1.94, respectively, and the frequency of quick‐circle was significantly higher in urine‐added water (Mann‐Whitney U test; *p* < 0.001).

### 3.5 Urine‐Somatic Index of Males, Non‐Ovulating Females, and Ovulating Females

**Figure 8:**
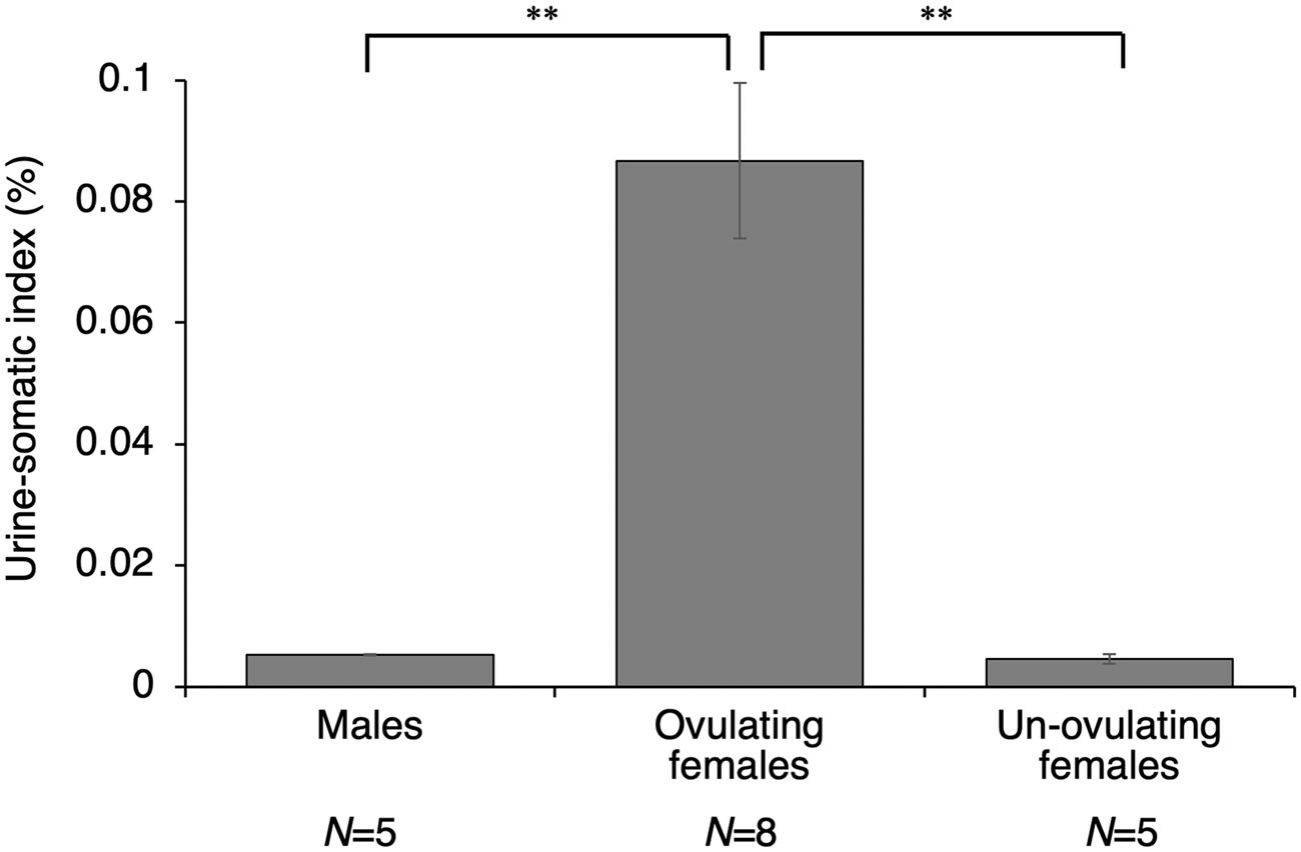
Urine‐somatic indices of males, ovulating females, and un‐ovulating females of medaka fish, *Oryzias latipes*. **p < 0.05 (Steel–Dwass test).

The urine weight (mean ± SEM; mg) of males, ovulating females, and non‐ovulating females were 0.01 ± 0.0018, 0. 27 ± 0.014, and 0.006 ± 0.0016, respectively. The urine‐somatic indices of males, ovulating females, and non‐ovulating females were 0.0052 ± 0.0001, 0.0867 ± 0.0128, and 0.0046 ± 0.0008, respectively (Figure 8). The Kruskal–Wallis test revealed significant differences (*p* = 0.001), with the urine‐somatic index in ovulating females being significantly greater than that in males (Steel‐Dwass test; *p* = 0.028) and non‐ovulating females (Steel‐Dwass test; *p* = 0.003).

## 4 Discussion

In the present study, we hypothesized that olfactory cues inducing male courtship behavior are released from ovulating female medaka, and attempted to verify this hypothesis by measuring the frequency of courtship behavior on males. It has been reported that male courtship behavior in medaka can be induced by olfactory and visual stimuli (Ono and Uematsu 1968a). Therefore, even if females release olfactory cues, the visual effects may obscure their impact. If olfactory stimulation induces male courtship behavior, the effect would be detected more clearly by counting the number of times the behavior occurs within as short a period as possible after exposure to the stimulation. In medaka, the time of the first appearance of each mating behavior from the encounter between male and female differs for each behavior, with following and positioning behavior occurring earlier and quick‐circle occurring later (Ono and Uematsu 1968b). In the present study, we measured the frequency of following and positioning behaviors for 10 min, and quick‐circle for 40 min after pairing. We also measured the time from when the male and female met until the first following behavior occurred.

The time it took males to first following to post‐spawning females tended to be longer than that for ovulating females. The frequency of males following, positioning, and quick‐circle behavior toward post‐spawning females was extremely low compared with their frequency toward ovulating females, and none of the six pairs observed reached contact/wrapping. This suggested that adult medaka males are more proactive in courtship with females capable of spawning. It was also shown that although males do following toward post‐spawning females, which tales some time, they rarely engaged in subsequent courtship behavior. Since Ono and Uematsu (1968a) have shown the importance of vision in medaka reproduction, we cannot exclude the possibility that the greater number of courtship behaviors shown for ovulating females in this study was due to visual factors, such as differences in the size of the female abdomen. In addition, the lack of any subsequent behavior after following the post‐spawning female may be due, in part, to the fact that the post‐spawning female did not show any reaction to the following by males. In contrast, Hayakawa et al. (2012) showed that the frequency of courtship behavior was reduced in males whose snouts were sealed with adhesives. Therefore, the low frequency of courtship behavior toward post‐spawning females in the present study may be due to the lack of olfactory stimulation from females. Behavioral observations toward post‐spawning females alone cannot indicate which influence caused the difference in the number of courtship behaviors by males. Therefore, we attempted to verify the hypothesis that olfactory cues induce courtship behavior in the present study by performing a stimulus‐water experiment using post‐spawning females.

When ovulating‐female water was used as the stimulus water, the time required for the first following (Kruskal‐Wallis test; *p* = 0.1716) and the frequencies of following (Kruskal‐Wallis test; *p* = 0.1141) and quick‐circle (Kruskal‐Wallis test; *p* = 0.128) were not significantly different from those in tap or male water. However, the averages of the following and quick‐circle were consistently 2.86 and 13.3 times higher, respectively in ovulating‐female water. In addition, the frequency of positioning was generally higher in ovulating‐female water than in tap water. This suggests that ovulating females may release olfactory cues that induce male courtship behavior, and that male's courtship behavior toward ovulating females is based on olfactory rather than visual cues. Hayakawa et al. (2012) conducted an experiment using male medaka with blocked nostrils and showed that removing olfactory stimuli inhibited spawning. Considering the results of the present study, those of Hayakawa et al. (2012) are thought to be due to males being unable to receive olfactory cues released by females. The frequency of male courtship behavior toward post‐spawning females in ovulating‐female water was lower than that toward ovulating females. This means that the effect of the olfactory cues in ovulating‐female water was lower than that of the olfactory cues emitted by real ovulating females. It is possible that ovulating females did not release chemical cues at all times but released higher levels when they detect the presence of adult males.

In medaka, various types of male courtship behavior (e.g., following, positioning, and quick‐circle) are observed, and these types change over time depending on the interaction with the female (Ono and Uematsu 1957). This suggests that olfactory cues emitted by ovulating females do not simply initiate male courtship behavior, but that the effect may be enhanced or the components or functions of olfactory cues may change depending on the interaction with the male. We investigated the frequency of courtship behavior in males toward post‐spawning females using stimulus water before quick‐circle (pre‐quick‐circle water) and after complete spawning (post‐spawning water) to investigate this possibility. In the experiments using ovulating‐female water, there was no significant increase in male following toward post‐spawning female, whereas in pre‐quick‐circle water, the frequency of males following was significantly higher than in tap water. This suggests that when ovulating female begin to receive courtship from male, she releases more olfactory cues that promote the males' following. On the other hand, the frequency of positioning and quick‐circle did not increase in pre‐quick‐circle water, suggesting that the chemical stimuli eliciting following, which are thought to be emitted from ovulating females, do not induce positioning or quick‐circle. On the other hand, male's quick‐circle to post‐spawning females was more frequently in post‐spawning water than in tap water. This suggests that as female continue to receive courtship behavior from males, she releases additional olfactory signals that at some stage induce the quick‐circle. In observations of wild medaka spawning behavior, male has been observed to perform quick‐circle female after she holding eggs on her abdomens (Kobayashi et al. 2012), and similar observations have also been reported in laboratory observations (Hayakawa et al. 2012). These observations are plausible if the olfactory cues that promote quick‐circle, released during the latter part of courtship behavior, remain around the female.

Sex pheromones are released in the urine of goldfish *Carassius auratus* goldfish (Sorensen et al. 1988; Appelt and Sorensen 2007) and salmonids (Scott et al. 1994; Vermeirssen et al. 1997; Yambem et al. 1999; Yambe 2009). In the present study, we hypothesized that ovulating medaka females secrete olfactory cues that induce courtship behavior in males through urine and investigated whether adding urine from ovulating females to tap water would change the courtship behavior of males toward post‐spawning females. In urine‐added water, the frequency of following and positioning toward post‐spawning females did not change, however, the frequency of quick‐circles increased significantly. This suggested that the urine of ovulating females contains olfactory cue that strongly induce the quick‐circle. Considering that the quick‐circle was promoted when post‐spawning water was used as the stimulus water, it is highly likely that the chemical cues contained in the post‐spawning water originate from urine.

On the other hand, the fact that the first step in a series of courtship behaviors, following, was not promoted by the urine of ovulating females suggests that the clues that males use to determine whether female is available to spawn are chemical signals released by a different pathway other than urine. In other words, ovulating females of medaka release two types of olfactory cues by different routes before and during courtship behavior with males, one of which is released before the start of courtship behavior to induce the male to male's following, and the other is released during courtship behavior via urine to promote the male's quick‐circle. Goldfish males are likely to detect androstenedione and 17α,20β‐dihydroxy‐4‐pregnen‐3‐one (17,20β‐P) only in close proximity to females, whereas sulfated 17,20β‐P is detected in distant urine patches (Sorensen et al. 1987, 1990, 2005; Scott and Ellis 2007). This suggests the existence of a dual pheromone system in goldfish, where preovulatory steroid pheromone components are not only spatially separated by urinary and gill emissions but are also perceived in different social contexts (Stacey 2024). In medaka, too, the olfactory stimuli inducing male following and quick‐circle are thought to be released from different pathways; the latter is likely to be released via urine, whereas the former may be released via a different pathway, such as the gills.

If ovulating female medakas release olfactory signals from urine, they may store more urine than usual in their bladders. In the present study, we compared the amount of urine in the bladders of ovulating female medaka with that of males and non‐ovulating females. The results showed that ovulating female medakas store more urine in their bladders than males and non‐ovulating females. This amount was more than ten times that of males and non‐ovulating females, indicating that they store a considerable amount of urine. The average urine weight of ovulating female was 0.27 mg, which is less than the amount of three ovulated eggs (diameter approximately 1.2 mm) of medaka, and is therefore considered to be an acceptable amount for medaka, which ovulate a dozen or so eggs at a time. This is the first study to demonstrate that females of teleost specifically store large amounts of urine in their bladders during ovulation to release olfactory cues. It is possible that ovulating female medaka use chemical signals in their urine to notify males of their courtship behavior. This point will be clarified in future studies that visualize the timing of urine excretion by females.

With the only obvious exception of the amino acid l‐kynurenine in the urine of masu salmon (*Oncorhynchus masou*) (Yambe et al. 2006), all sex pheromones described in bony fishes are reproductive hormones, their precursors, and metabolites (Stacey 2024). In particular, 17,20β‐P secreted from the female ovary during oocyte maturation (Nagahama 1997), and prostaglandin (PG) secreted during ovulation (Takahashi et al. 2018), and their metabolites are known to act as pheromones to induce male sexual behavior in many fish species (Stacey 2024). In medaka, 17,20β‐P and PGE_2_ play an important role in oocyte maturation and ovulation, respectively (Shibata et al. 2011; Hagiwara et al. 2014; Takahashi and Ogiwara 2025). The peak of 17,20β‐P production in medaka ovarian follicles is thought to occur 10 h before the expected time of spawning (8 h before the expected time of ovulation) (Shibata et al. 2011). In the present study, we used females that had already ovulated (presumably several hours after ovulation), so it is likely that the blood concentrations of 17, 20β‐P were low at the time of the experiment. On the other hand, PGs are not only secreted during ovulation (Takahashi et al. 2018), but can also be produced in response to the presence of ovulated oocytes in the ovaries (Sorensen et al. 2018), so it is possible that PGs or their metabolites were still at high concentrations in the blood at the time of the experiment. Furthermore, it has also been shown experimentally that administration of PGF_2α_ (not a natural ovulation inducer for medaka) to preovulatory females induces courtship behavior in male medakas (Oshima et al. 2003; Nakayama et al. 2004). From the above, it is highly likely that at least one of the olfactory stimulants that induces male mating behavior in medaka is PG or its metabolites. Future studies should clarify whether PGE_2_ excreted by female medaka has the effect of inducing male courtship behavior, especially following, and if so, how it is excreted from the body.

## 5 Conclusion

In the present study, we were able to demonstrate for the first time in medaka that ovulating female release olfactory cues that induce male courtship behavior using the post‐spawning females in which males do not show courtship behavior. At least two types of olfactory cues having different functions thought to be released through different routes, one of them has the function of indicating the female's egg‐laying ability (ovulating), and the other has the function of increasing the male's motivation for courtship behavior toward female. The latter olfactory cue is contained in the urine of ovulating females, and we confirmed that ovulating female medaka store more than ten times the amount of urine in their bladders than non‐ovulating females. The results of the present study predict that female medaka receiving courtship behavior from males release urine at certain times to increase the male's motivation for the behavior.

## Conflicts of Interest

The authors declare no conflicts of interest.

## Data Availability

Data will be made available on request.

## References

[jez70017-bib-0001] Anholt, R. R. H. , P. O'Grady , M. F. Wolfner , and S. T. Harbison . 2020. “Evolution of Reproductive Behavior.” Genetics 214: 49–73. 31907301 10.1534/genetics.119.302263 PMC6944409

[jez70017-bib-0002] Appelt, C. W. , and P. W. Sorensen . 2007. “Female Goldfish Signal Spawning Readiness by Altering When and Where They Release a Urinary Pheromone.” Animal Behaviour 74: 1329–1338.

[jez70017-bib-0003] Ashouri, S. , J. P. Da Silva , A. Canário , and P. C. Hubbard . 2023. “Bile Acids as Putative Social Signals in Mozambique Tilapia (*Oreochromis mossambicus*).” Physiology & Behavior 272: 114378. 37858914 10.1016/j.physbeh.2023.114378

[jez70017-bib-0004] Egami, N. , and M. Nambu . 1961. “Factors Initiating Mating Behavior and Oviposition in the Fish, *Oryzias latipes* .” Journal of the Faculty of Science, University of Tokyo Section Ⅳ, Zoology 9: 263–278.

[jez70017-bib-0005] Grant, J. W. A. , and L. D. Green . 1996. “Mate Copying Versus Preference for Actively Courting Males by Female Japanese Medaka (*Oryzias latipes*).” Behavioral Ecology 7: 165–167.

[jez70017-bib-0006] Hagiwara, A. , K. Ogiwara , Y. Katsu , and T. Takahashi . 2014. “Luteinizing Hormone‐Induced Expression of Ptger4b, a Prostaglandin E2 Receptor Indispensable for Ovulation of the Medaka *Oryzias latipes*, is Regulated by a Genomic Mechanism Involving Nuclear Progestin Receptor1.” Biology of Reproduction 90: 1–14. 10.1095/biolreprod.113.115485 24790162

[jez70017-bib-0007] Hayakawa, Y. , S. Takita , K. Kikuchi , A. Yoshida , and M. Kobayashi . 2012. “Involvement of Olfaction in Spawning Success of Medaka *Oryzias latipes* .” Japanese Journal of Ichthyology 59: 111–124.

[jez70017-bib-0008] Kobayashi, M. , T. Yoritsune , S. Suzuki , et al. 2012. “Reproductive Behavior of Wild Medaka in an Outdoor Pond.” Nippon Suisan Gakkaishi 78: 922–933.

[jez70017-bib-0009] Kondo, Y. , M. Kohda , and S. Awata . 2025a. “Male Medaka Continue to Mate With Females Despite Sperm Depletion.” Royal Society Open Science 12: 241668. 39780977 10.1098/rsos.241668 PMC11706666

[jez70017-bib-0010] Kondo, Y. , K. Okamoto , Y. Kitamukai , Y. Koya , and S. Awata . 2025b. “Medaka (*Oryzias latipes*) Initiate Courtship and Spawning Late at Night: Insights From Field Observations.” PLoS One 20, no. 2: e0318358. 39937747 10.1371/journal.pone.0318358 PMC11819472

[jez70017-bib-0011] Maruska, K. P. , and J. M. Butler . 2021. “Reproductive‐ and Social‐State Plasticity of Multiple Sensory Systems in a Cichlid Fish.” Integrative and Comparative Biology 61: 249–268. 33963407 10.1093/icb/icab062

[jez70017-bib-0012] Miranda, A. , O. G. Almeida , P. C. Hubbard , E. N. Barata , and A. V. M. Canário . 2005. “Olfactory Discrimination of Female Reproductive Status by Male Tilapia (*Oreochromis mossambicus)* .” Journal of Experimental Biology 208: 2037–2043. 15914647 10.1242/jeb.01584

[jez70017-bib-0013] Nagahama, Y. 1997. “17α,20β‐Dihydroxy‐4‐pregnen‐3‐one, a Maturation‐Inducing Hormone in Fish Oocytes: Mechanisms of Synthesis and Action.” Steroids 62: 190–196. 9029736 10.1016/s0039-128x(96)00180-8

[jez70017-bib-0014] Nakayama, K. , Y. Oshima , T. Yamaguchi , et al. 2004. “Fertilization Success and Sexual Behavior in Male Medaka, *Oryzias latipes*, Exposed to Tributyltin.” Chemosphere 55: 1331–1337. 15081776 10.1016/j.chemosphere.2003.11.050

[jez70017-bib-0015] Ono, Y. , and T. Uematsu . 1957. “Mating Ethogram in *Oryzias latipes* .” Journal of the Faculty of Science, Hokkaido University Series VI Zoology 13: 197–202.

[jez70017-bib-0016] Ono, Y. , and T. Uematsu . 1968a. “Experimental Analysis of the Sign Stimuli in the Mating Behavior in *Oryzias latipes* .” Japanese Journal of Ecology 18: 65–74.

[jez70017-bib-0017] Ono, Y. , and T. Uematsu . 1968b. “Sequence of the Mating Activities in *Oryzias latipes* .” Japanese Journal of Ecology 18: 1–10.

[jez70017-bib-0018] Oshima, Y. , I. J. Kang , M. Kobayashi , K. Nakayama , N. Imada , and T. Honjo . 2003. “Suppression of Sexual Behavior in Male Japanese Medaka (*Oryzias latipes*) Exposed to 17Β‐Estradiol.” Chemosphere 50: 429–436. 12656264 10.1016/s0045-6535(02)00494-0

[jez70017-bib-0019] Scott, A. P. , and T. Ellis . 2007. “Measurement of Fish Steroids in Water—A Review.” General and Comparative Endocrinology 153: 392–400. 17188270 10.1016/j.ygcen.2006.11.006

[jez70017-bib-0020] Scott, A. P. , N. R. Liley , and E. L. M. Vermeirssen . 1994. “Urine of Reproductively Mature Female Rainbow Trout, *Oncorhynchus mykiss* (Walbaum), Contains a Priming Pheromone Which Enhances Plasma Levels of Sex Steroids and Gonadotrophin II in Males.” Journal of Fish Biology 44: 131–147.

[jez70017-bib-0021] Shibata, N. , N. Nakamoto , Y. Shibata , and Y. Nagahama . 2011. “Endocrine regulation of oogenesis in the medaka, *Oryzias latipes* .” In Medaka: A Model for Organogenesis, Human Disease, and Evolution, edited by K. Naruse , N. Tanaka , and H. Takeda , 269–285. Springer.

[jez70017-bib-0022] Sorensen, P. , T. Hara , N. Stacey , and J. Dulka . 1990. “Extreme Olfactory Specificity of Male Goldfish to the Preovulatory Steroidal Pheromone 17Α,20Β‐Dihydroxy‐4‐Pregnen‐3‐One.” Journal of Comparative Physiology A 166: 373–383.

[jez70017-bib-0023] Sorensen, P. W. , C. Appelt , N. E. Stacey , F. W. Goetz , and A. R. Brash . 2018. “High Levels of Circulating Prostaglandin F_2_α Associated With Ovulation Stimulate Female Sexual Receptivity and Spawning Behavior in the Goldfish (*Carassius auratus*).” General and Comparative Endocrinology 267: 128–136. 29940184 10.1016/j.ygcen.2018.06.014

[jez70017-bib-0024] Sorensen, P. W. , T. J. Hara , and N. E. Stacey . 1987. “Extreme Olfactory Sensitivity of Mature and Gonadally‐Regressed Goldfish to a Potent Steroidal Pheromone, 17Α,20Β‐Dihydroxy‐4‐Pregnen‐3‐One.” Journal of Comparative Physiology A 160: 305–313.

[jez70017-bib-0025] Sorensen, P. W. , T. J. Hara , N. E. Stacey , and F. W. Goetz . 1988. “F‐Prostaglandins Function as Potent Olfactory Stimulants That Comprise the Postovulatory Female Sex Pheromone in Goldfish.” Biology of Reproduction 39: 1039–1050. 3219377 10.1095/biolreprod39.5.1039

[jez70017-bib-0026] Sorensen, P. W. , M. Pinillos , and A. P. Scott . 2005. “Sexually Mature Male Goldfish Release Large Quantities of Androstenedione Into the Water Where It Functions as a Pheromone.” General and Comparative Endocrinology 140: 164–175. 15639144 10.1016/j.ygcen.2004.11.006

[jez70017-bib-0027] Stacey, N. E. 2024. “Hormonally Derived Sex Pheromones in Fishes.” In Hormones and Reproduction of Vertebrates, edited by D. O. Norris and K. H. Lopez , 1, *Second Edition* , 217–316. Elsevier Inc.

[jez70017-bib-0028] Takahashi, T. , A. Hagiwara , and K. Ogiwara . 2018. “Prostaglandins in Teleost Ovulation: A Review of the Roles With a View to Comparison With Prostaglandins in Mammalian Ovulation.” Molecular and Cellular Endocrinology 461: 236–247. 28919301 10.1016/j.mce.2017.09.019

[jez70017-bib-0029] Takahashi, T. , and K. Ogiwara . 2025. “An Attempt to Search for the Common Cellular Mechanism of Ovulation Across All Metazoans.” Reproduction (Cambridge, England) 169: e240184. 39441770 10.1530/REP-24-0184

[jez70017-bib-0030] Vermeirssen, E. L. M. , A. P. Scott , N. R. Liley , and E. Vermeirssen . 1997. “Female Rainbow Trout Urine Contains a Pheromone Which Causes a Rapid Rise in Plasma 17,20β‐dihydroxy‐4‐pregnen‐3‐one Levels and Milt Amounts in Males.” Journal of Fish Biology 50: 107–119.

[jez70017-bib-0031] Yabuki, Y. , T. Koide , N. Miyasaka , et al. 2016. “Olfactory Receptor for Prostaglandin F_2_α Mediates Male Fish Courtship Behavior.” Nature Neuroscience 19: 897–904. 27239939 10.1038/nn.4314

[jez70017-bib-0032] Yambe, H. 2009. “Studies on Sex Pheromones in Salmonids.” Nippon Suisan Gakkaishi 75: 648–651.

[jez70017-bib-0033] Yambe, H. , S. Kitamura , M. Kamio , et al. 2006. “L‐Kynurenine, an Amino Acid Identified as a Sex Pheromone in the Urine of Ovulated Female Masu Salmon.” Proceedings of the National Academy of Sciences 103: 15370–15374. 10.1073/pnas.0604340103 PMC1622830 17030810

[jez70017-bib-0034] Yambem, H. , M. Shindo , and F. Yamazaki . 1999. “A Releaser Pheromone That Attracts Males in the Urine of Mature Female Masu Salmon.” Journal of Fish Biology 55: 158–171.

